# The role of clusterin and the variant rs11136000_T throughout the spectrum of Alzheimer’s disease

**DOI:** 10.1002/alz.089488

**Published:** 2025-01-03

**Authors:** Marina Tedeschi Dauar, Cynthia Picard, Anne Labonte, Sylvia Villeneuve, Judes Poirier, PREVENT‐AD Research Group

**Affiliations:** ^1^ Douglas Mental Health Research Centre, Montreal, QC Canada; ^2^ CAPES Foundation, Ministry of Education of Brazil, Brasilia Brazil; ^3^ McGill University, Montreal, QC Canada; ^4^ Centre for Studies on Prevention of Alzheimer’s disease (StoP‐AD Centre), Douglas Mental Health Institute, Montreal, QC Canada; ^5^ Department of Psychiatry, McGill University, Montréal, QC Canada

## Abstract

**Background:**

Clusterin is a major cholesterol transporter in the central nervous system (CNS) and different SNPs in the *CLU* gene have been associated with Alzheimer’s disease (AD) risk. The rs11136000_T variant in the *CLU* gene has been shown to decrease the risk of AD. In this work, we investigate the role of the CLU rs11136000_T protective variant and of the clusterin protein throughout different phases of the AD spectrum.

**Method:**

Levels of clusterin, Tau, phospho(181) Tau (p‐tau), and Aß were measured using ELISA and synaptic proteins were measured using immunoprecipitation followed by mass spectroscopy in the CSF of cognitively unimpaired individuals. Flortaucipir PET was used to measure tau deposition and 18F‐NAV4694 PET was used to measure amyloid burden in the same subjects. ELISA was used to measure clusterin levels and RT‐PCR was used to measure mRNA levels in cortical areas of autopsied‐confirmed controls and AD subjects. Genotype was performed using Illumina Infinium Omni2.5M‐8 microarray.

**Result:**

There was no effect of the protective genotype on the levels of PET biomarkers, CSF biomarkers or synaptic proteins in cognitively unimpaired individuals. CSF clusterin levels were positively associated with Aß (p = 0.021), total‐tau (p<0.001), p‐tau (p<0.001), synaptotagmin (p<0.001), SNAP 25 (p = 00006), GAP 43 (p = 0.00055) and neurogranin (p = 0.0086) in the CSF and with amyloid deposition in the hippocampus (p = 0.018) and tau retention in the entorhinal cortex (p = 0.0082) and temporal pole (p = 0.0124) in cognitively unimpaired individuals at high risk for AD. Clusterin is increased in the brain of individuals who are homozygous for the CLU_T variant (p = 0.04) and in AD patients compared to age‐matched controls (p = 0.024). mRNA levels are increased in individuals homozygous for the CLU_T variant (p = 0.003), but there are no significant differences in AD patients compared to controls (p = 0.07).

**Conclusion:**

Our work shows significant correlation between CSF clusterin and the main AD biomarkers (PET and CSF) and synaptic proteins before disease onset, in subjects at high risk for the disease. In the later stage of the disease, clusterin protein and *CLU* mRNA levels are increased as a function of genotype and disease status, suggesting that *CLU* may play a protective role by increases in gene expression in the CNS.

